# Shared Genetic Liability Between Major Depressive Disorder and Atopic Diseases

**DOI:** 10.3389/fimmu.2021.665160

**Published:** 2021-09-08

**Authors:** Hongbao Cao, Sheng Li, Ancha Baranova, Fuquan Zhang

**Affiliations:** ^1^Department of Psychiatry, First Hospital/First Clinical Medical College of Shanxi Medical University, Taiyuan, China; ^2^School of Systems Biology, George Mason University, Fairfax, VA, United States; ^3^Institute of Systems Medicine, Chinese Academy of Medical Sciences & Peking Union Medical College, Beijing, China; ^4^Suzhou Institute of Systems Medicine, Suzhou, China; ^5^Research Centre for Medical Genetics, Moscow, Russia; ^6^Department of Psychiatry, The Affiliated Brain Hospital of Nanjing Medical University, Nanjing, China; ^7^Institute of Neuropsychiatry, The Affiliated Brain Hospital of Nanjing Medical University, Nanjing, China

**Keywords:** major depressive disorder, Mendelian randomization, meta-analyses, asthma, atopic diseases

## Abstract

**Objectives:**

Deciphering the genetic relationships between major depressive disorder (MDD) and atopic diseases (asthma, hay fever, and eczema) may facilitate understanding of their biological mechanisms as well as the development of novel treatment regimens. Here we tested the genetic correlation between MDD and atopic diseases by linkage disequilibrium score regression.

**Methods:**

A polygenic overlap analysis was performed to estimate shared genetic variations between the two diseases. Causal relationships between MDD and atopic diseases were investigated using two-sample bidirectional Mendelian randomization analysis. Genomic loci shared between MDD and atopic diseases were identified using cross-trait meta-analysis. Putative functional genes were evaluated by fine-mapping of transcriptome-wide associations.

**Results:**

The polygenic analysis revealed approximately 15.8 thousand variants causally influencing MDD and 0.9 thousand variants influencing atopic diseases. Among these variants, approximately 0.8 thousand were shared between the two diseases. Mendelian randomization analysis indicates that genetic liability to MDD has a causal effect on atopic diseases (b = 0.22, p = 1.76 × 10^-6^), while genetic liability to atopic diseases confers a weak causal effect on MDD (b = 0.05, p = 7.57 × 10^-3^). Cross-trait meta-analyses of MDD and atopic diseases identified 18 shared genomic loci. Both fine-mapping of transcriptome-wide associations and analysis of existing literature suggest the estrogen receptor β-encoding gene *ESR2* as one of the potential risk factors for both MDD and atopic diseases.

**Conclusion:**

Our findings reveal shared genetic liability and causal links between MDD and atopic diseases, which shed light on the phenotypic relationship between MDD and atopic diseases.

## Introduction

Mental disorders confer a heavy burden on society (1). Major depressive disorder (MDD), the most prevalent mental disorder accompanied by considerable morbidity, mortality, and risk of suicide, is characterized by persistent low mood (2). MDD and depressive symptoms have close associations with certain physical conditions. Generally speaking, long-term depression adds to the risk for somatic illness, and, vice versa, chronic somatic diseases are frequently accompanied by depression (3). When comorbid with other ailments, for example, atopic diseases (ADs), MDD produces worse clinical outcomes and incurs higher healthcare costs.

ADs are driven by the dysfunction of the immune system. Three kinds of common ADs, namely, asthma, hay fever (allergic rhinitis), and eczema (atopic dermatitis), may coexist in the same individuals (4). Asthma, a chronic airway disease that is common worldwide, is characterized by coughing, wheezing, shortness of breath, and/or chest tightness due to increased airway reactivity, inflammation, and/or mucus production. In 2015, asthma affected 358 million people globally and caused about 400,000 deaths (5). Allergic rhinitis is an inflammatory disease characterized by nasal congestion, rhinorrhea, sneezing, and/or nasal itching. Allergic rhinitis is one of the most common diseases in adults (20%~30%), and the most common chronic disease in children (up to 40%) in the United States (6). Eczema is an inflammatory skin disease that is caused by a dysfunction of a skin barrier followed by aberrant inflammation/immune responses; this disease is affecting 5% of the population worldwide (7). Together, symptoms of ADs significantly impair quality of life and impose a heavy cost on society. Common comorbidities of MDD with ADs have been documented previously (8–12). Specifically, allergic rhinitis has been shown to have a positive association with MDD (odds ratio: 1.24) (8). In patients with asthma, the hazard ratio of MDD increases by 35%, and MDD patients show about 25% increased hazard ratio for being affected by asthma (9). Atopic eczema is also associated with an increased incidence of new depression (hazard ratio: 1.14) (10).

Although previous studies have detected associations between MDD and ADs, several key questions remain pending: 1) to what extent may the two conditions share genetic components? 2) Are the phenotypic associations mediated by genetic variations? 3) What molecular and cellular mechanisms underline these associations?

Genetic relationships between two traits are commonly quantified by genetic correlation coefficients. The sign of the correlation coefficient indicates directions of the shared genetic effects. When dealing with mixtures of effect directions across shared genetic variants, genetic correlation analyses may be underpowered (13). A polygenic overlap was recently proposed to measure the fraction of genetic variants causally associated with both traits over the total number of causal variants across a pair of traits involved (13).

Mendelian randomization (MR) is an analytic framework that utilizes genetic variants as instrumental variables to test for causative association between an exposure and an outcome (14). Recently, a general type of SMR (GSMR) had been developed by leveraging power from multiple genetic variants to account for linkage disequilibrium (LD) between the variants (15).

Recently, Zhu et al. reported a causal effect of MDD on asthma and identified 10 loci shared by asthma and MDD by cross-trait meta-analysis (16). The GWAS dataset for MDD, however, did not include the 23andMe samples. We set on taking this line of investigation further, by both utilizing a larger MDD dataset and including two other ADs related to asthma, namely, allergic rhinitis and atopic dermatitis. Asthma, allergic rhinitis, and atopic dermatitis genetically correlate with each other and are often comorbid (17). The genetic liability to MDD may confer a causal effect on all of these ADs. Dissection of this shared genetic liability may deliver novel insights into the pathophysiology of both MDD and ADs.

## Methods

### GWAS Summary Datasets and Quality Control

This study relied on both de-identified publicly available summary-level GWAS data and the pre-approval 23andMe dataset. The resultant MDD dataset included 135,458 cases and 344,901 healthy controls (18), and the AD dataset included 96,794 cases and 145,775 healthy controls (19). For the inclusion of each dataset, both bi-allelic SNPs and imputation INFO above 0.80 were required. Each SNP was compared between the two datasets, and SNPs with conflicting alleles were excluded. If an SNP was mapped to opposite strands in the two datasets, alleles of this SNP in the second dataset were flipped, and the effect direction was reversed.

### Genetic Correlation and Polygenic Overlap Analysis

GWAS summary results were utilized to analyze the genetic correlation of MDD with ADs by LD score regression software (LDSC, v1.0.1) (20, 21). A polygenic overlap was analyzed by MiXeR v1.2 using default parameters (13). Using GWAS summary statistics, MiXeR quantifies the polygenic overlap irrespective of the genetic correlation between traits. Based on the univariate causal mixture model (22), MiXeR builds four bivariate normal distributions, with two causal components for variants specific to each trait, one causal component for variants affecting both traits, and a null component for variants with no effect on either trait. The likelihood function of the observed signed test statistics (GWAS Z-scores) is produced from the prior distribution of genetic effects, incorporating effects of the LD structure, sample size, minor allele frequency (MAF), cryptic relationships, and sample overlap. The summary statistics are used to estimate the parameters of the mixture model by optimization of the likelihood function. The number of causal variants reported by the software is 22.6% of the total estimated variants, which account for 90% of SNP heritability for each trait.

### MR Analysis

Bidirectional causal associations between MDD and ADs were inferred using GSMR v1.0.9 (15). Instrumental variants were selected based on default p ≤ 5×10^-8^. It is well accepted that pleiotropy is a potential source of bias and an inflated estimation in an MR analysis (23). In GSMR, the HEIDI-outlier statistical approach allows the detection and elimination of genetic instruments with apparent pleiotropic effects on both risk factors and disease (15, 24). It was suggested that genetic correlation may confound Mendelian randomization estimates (25). To examine this possibility, we performed a latent causal variable model (LCV) analysis between MDD and ADs (26). The LCV framework utilizes the genetic causality proportion (GCP) to quantify the partial causality of trait 1 on trait 2. The GCP ranges from 0 (no partial genetic causality) to 1 (full genetic causality). A high value of GCP indicates a causal effect of interventions targeting trait 1 on trait 2.

### Cross-Trait Meta-Analysis

A cross-trait meta-analysis of the MDD and the ADs was executed by the subset-based fixed-effect method ASSET v2.4.0, which permits the characterization of each SNP with respect to its pattern of effects on multiple phenotypes (27). For each assessed variant, this type of analysis returns a p-value for the best subset containing the studies contributing to the overall association signal. The meta-analysis pools the effect of a given SNP across K studies, weighting the effects by the size of the respective study. After subset-based meta-analysis, SNP-related findings were considered statistically significant, if two-tailed p values were lower than 5 × 10^-8^. In the meta-analysis results, functional annotation and gene-mapping of variants and identifying LD-independent genomic regions were performed on a FUMA platform (28). Firstly, independent significant SNPs (IndSigSNPs) were identified based on their p-value being genome-wide significant (p ≤ 5.0 × 10^−8^) and independent of each other (r^2^ < 0.6). Secondly, lead SNPs were identified as a subset of the independent significant SNPs that were in LD with each other at r^2^ < 0.1 within a 250-kb window. The gene-based association for the meta-analysis of MDD and ADs was conducted using MAGMA (29).

To ensure that sample overlap did inflate estimates of genetic overlap between MDD and ADs, λmeta statistics, which use effect size concordance to detect sample overlap or heterogeneity, were calculated (30). Under the null hypothesis, λmeta equals 1 when the pair of cohorts are completely independent. When there are overlapping samples, λmeta is less than 1.

### Fine-Mapping of TWAS Associations

To prioritize putatively causal genes, fine-mapping of causal gene sets (FOCUS v0.6.10) (31) to the meta-analysis result of MDD and ADs was performed in four relevant tissues, including the brain, whole blood, lung, and skin. Using FOCUS, predicted expression correlations were modeled and posterior inclusion probabilities (PIP) are assigned to genes within each transcriptome-wide association study (TWAS) region in the relevant tissue types. A multi-tissue eQTL reference weight database from the software was used as eQTL weights, while LD information from LDSC was used as a reference. Multiple-testing correction was used to account for all gene–tissue pairs based on Benjamini–Hochberg adjusted TWAS p-values (FDR < 0.05).

### Knowledge-Based Analysis

GWAS results, including meta-analysis, were obtained for depression (major depressive disorder and depressive symptoms) and for ADs from the GWAS Catalog database (access date: April 17, 2020) (32). We explore whether the genes shared by MDD and ADs have been identified in previous genome-wide association studies. Protein–protein interaction analysis was conducted using STRING v11 (33). Enrichment of the 27 genes in the GWAS catalog reported genes was analyzed using FUMA (28).

All the statistical analyses were conducted in R 3.6.1 or Python 3.7 environment. A detailed description of the methods is provided in the **Supplementary File**.

## Results

### Genetic Correlation and Polygenic Overlap Analysis

**Figure 1:**
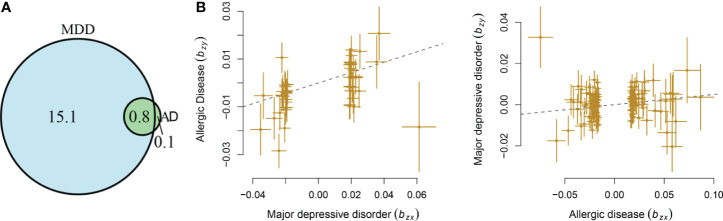
Shared causal variants and causal associations between MDD and ADs. **(A)** Venn diagrams of unique and shared polygenic components at the causal level, showing a polygenic overlap between MDD and ADs. The numbers indicate the estimated quantity of causal variants (in thousands) per component, explaining 90% of SNP heritability in each phenotype. The size of the circles reflects the degree of polygenicity. **(B)** Causal associations between MDD and ADs. The lines denote effect sizes **(B)**. The left panel denotes the causal effect of MDD on ADs. The left panel denotes the causal effect of ADs on MDD.

MDD displayed a significant genetic correlation with ADs (r = 0.18, s.e. = 0.03, p = 1.04 × 10^-9^). The LD score intercept did not deviate from zero (0.017). The polygenic analysis highlighted approximately 15.8 thousand variants causally influencing MDD and 0.9 thousand variants influencing ADs. Among these variants, approximately 0.8 thousand variants were shared between the two diseases (**Figure 1A**). MDD has much larger numbers of causal variants than ADs, indicating a higher polygenic property of MDD.

### MR Analysis

Mendelian randomization analysis indicated that genetic liability to MDD has a causal effect on ADs (b = 0.22, s.e. = 0.05, OR = 1.25, 95%CI: 1.13–1.37, p = 1.76 × 10^-6^), with 45 independent instrumental variants being involved. The genetic liability of ADs conferred a causal effect on MDD (b = 0.05, s.e. = 0.02, OR = 1.05, 95%CI: 1.01–1.09, p = 7.57 × 10^-3^), with 115 independent instrumental variants being involved (**Figure 1B**). The LCV analysis showed that GCP was 0.49 (0.32), supporting a causal effect of genetic liability to MDD on ADs.

### Cross-Trait Meta-Analysis

**Figure 2:**
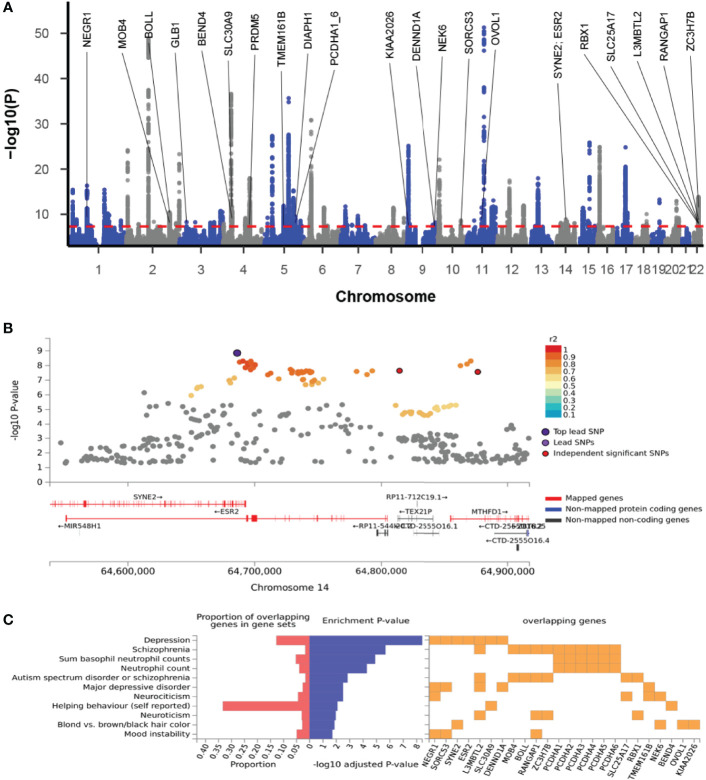
Cross-trait meta-analysis of MDD and ADs. **(A)** Manhattan plot of meta-analysis of MDD with ADs. The x-axis is the chromosomal position of SNPs, and the y-axis is the significance of the SNPs (-log_10_P). Protein-coding genes containing or adjacent to independent significant SNPs shared by two traits were annotated. PCDHA1_6: *PCDHA1*, *PCDHA2*, *PCDHA3*, *PCDHA4*, *PCDHA5*, and *PCDHA6*. **(B)** The 14q23 locus containing the *ESR2* gene. Each SNP is colored based on the highest r^2^ to one of the independent significant SNPs. **(C)** Enrichment of the 27 protein-coding genes in GWAS catalog gene sets.

The cross-trait meta-analysis of MDD and ADs revealed the involvement of 103 loci, 470 significant independent SNPs (IndSigSNPs), and 141 lead SNPs, including 44 pleiotropic IndSigSNPs located in 18 loci (associated with both traits) (**Figure 2A**, **Table 1** and **Supplementary Tables 1, 2**). The 14q23 locus is shown in **Figure 2B**. A total of 82 pleiotropic protein-coding genes were identified, including 27 protein-coding genes implicated by the pleiotropic IndSigSNPs and another 55 protein-coding genes implicated by SNPs tagged by IndSigSNPs (**Supplementary Table 3**). The gene-based association for the meta-analysis of MDD and ADs identified a total of 273 significant genes at the threshold of 2.70 × 10^-6^ (Bonferroni correction, 0.05/18,545) (**Supplementary Table 4**). Compared with SNP-based analysis, an additional 63 genes were identified by the gene-based analysis, including DRD2. The λmeta value was at 1.18 for datasets between MDD and ADs, indicating no significant overlap between MDD and AD GWAS samples. Quantile–quantile (QQ) plots to display the observed meta-analysis statistics versus the expected statistics under the null model of no associations in the -log10(p) scale are shown in **Supplementary Figure 1**.

**Table 1:** Genomic loci shared between MDD and ADs.

SNP	Chr : BP	P	Start : End	Genes
rs10789340	1:72940273	4.85×10^-17^	72512988:72958905	**NEGR1**; RPL31P12
rs700646	2:198608511	3.80×10^-11^	198148191:198954774	**MOB4**; **BOLL**; AC011997.1
rs11927929	3:33087057	5.46×10^-9^	33068268:33126972	**GLB1**
rs34215985	4:42047778	2.07×10^-12^	41882601:42187640	RP11-457P14.5; RP11-457P14.6; **SLC30A9**; **BEND4**
rs71600495	4:121628028	1.57×10^-8^	121625080:121655414	**PRDM5**
rs247910	5:87630769	1.41×10^-12^	87437079:88065637	**TMEM161B**; TMEM161B-AS1; LINC00461; CTC-467M3.1
rs1363105	5:103917790	1.80×10^-10^	103671867:104082179	RP11-6N13.1
rs10060640	5:140211226	7.62×10^-9^	140024042:140222641	**PCDHA1; PCDHA2; PCDHA3; PCDHA4; PCDHA6; PCDHA5**
rs3844598	5:140992235	3.14×10^-10^	140893490:141032603	**DIAPH1**
rs11135349	5:164523472	2.71×10^-9^	164465319:164678946	CTC-340A15.2
rs2064219	6:27376001	3.07×10^-10^	25684606:29607101	MCFD2P1
rs144829310	9:6208030	7.58×10^-26^	5609742:6621027	AK4P4; **KIAA2026**
rs549779	9:126613028	2.62×10^-8^	126452936:126714710	**DENND1A**
rs10818936	9:127006346	3.82×10^-8^	126999153:127144622	**NEK6**
rs61867293	10:106563924	2.60×10^-9^	106529451:106830537	**SORCS3**
rs479844	11:65551957	3.64×10^-12^	65401336:65641033	**OVOL1**
rs915057	14:64686207	1.42×10^-9^	64649894:64877135	**SYNE2; ESR2**
rs136402	22:41598933	1.51×10^-14^	41085969:42216326	**SLC25A17**; **RBX1**; Y_RNA; RP11-12M9.4; RP1-85F18.5; **L3MBTL2**; **RANGAP1**; **ZC3H7B**

Chr, chromosome; BP, base position. Protein-coding genes are shown in bold.

### Fine-Mapping of TWAS Associations

**Figure 3:**
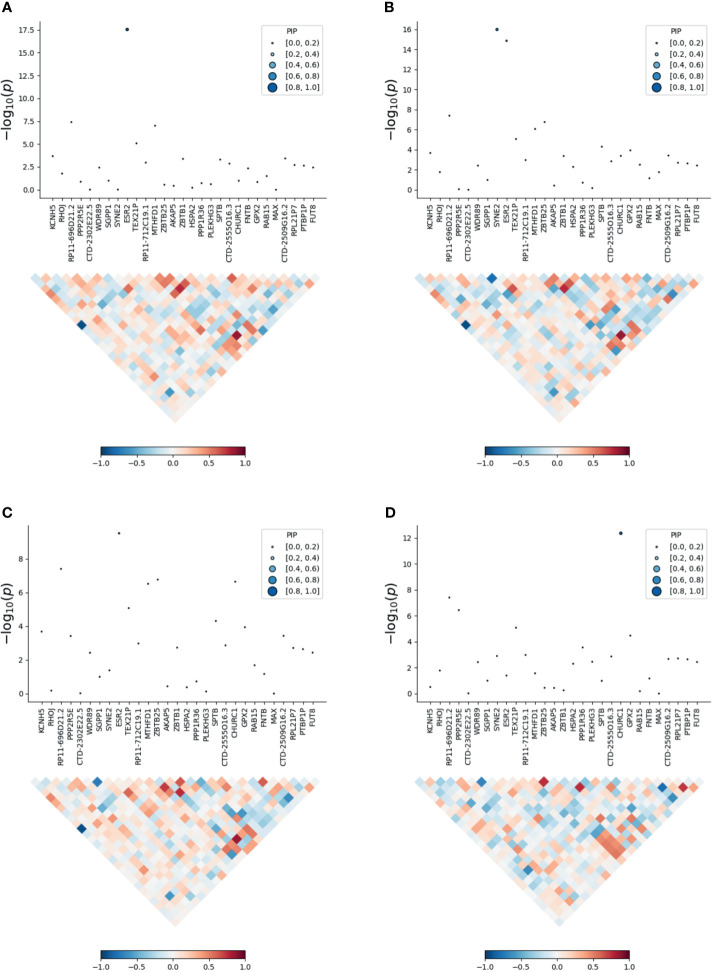
Transcriptome-wide association study of the meta-analysis of MDD and ADs. **(A)** skin; **(B)** blood; **(C)** lung; **(D)** brain. Within each panel, the top part is the transcriptome-wide association signal indicating strength of the predicted expression association with trait, and the bottom part is the induced correlation of the predicted expression.

To prioritize putatively causal genes, we used the fine-mapping of TWAS associations. A total of 126 gene–tissue pairs were identified between the 82 genes and the four tissues, with 36 genes being associated with two or more tissues (**Supplementary Table 5**). A total of 31 gene–tissue pairs were in the credible sets. Fifteen genes associated with three or more tissues are listed in **Table 2**. However, most genes in **Table 2** had low PIP. Of note, the *ESR2* gene was associated with three tissues (skin, lung, and blood) with relatively high posterior probability (**Figure 3**).

**Table 2:** TWAS analysis in the four tissues.

Gene	GWAS P	Chr : Start-End	Tissue	Brain Z (PIP)	Blood Z (PIP)	Lung Z (PIP)	Skin Z (PIP)
SLC30A9	2.07×10^-12^	4:41992489-42092474	Brain, blood, lung	-6.08 (0.313)	-1.83 (<0.01)	3.72 (<0.01)	
NDUFA2	2.25×10^-6^	5:140018325-140027370	Brain, blood, skin	6.27 (0.941)	4.28 (0.011)		-2.87 (<0.01)
FCHSD1	5.99×10^-8^	5:141018869-141030986	Brain, lung, blood	5.64 (0.807)	1.86 (<0.01)	2.42 (<0.01)	
PCDHA7	7.62×10^-9^	5:140213969-140391929	Lung, brain, skin	-5.02 (0.150)		-5.18 (0.317)	-3.65 (0.013)
WDR55	2.24×10^-6^	5:140044261-140053709	Skin, blood, lung, brain	-1.56 (<0.01)	-4.65 (0.026)	-1.86 (<0.01)	-4.69 (0.059)
IK	2.25×10^-6^	5:140026643-140042064	Blood, skin, lung, brain	-3.67 (<0.01)	4.52 (0.034)	-3.7 (<0.01)	-3.37 (<0.01)
TMCO6	2.25×10^-6^	5:140019012-140024993	Blood, skin, lung, brain	-1.61 (<0.01)	-4.27 (<0.01)	-3.41 (<0.01)	-2.98 (<0.01)
ZMAT2	2.51×10^-6^	5:140078265-140086248	Lung, blood, skin, brain	-3.5 (<0.01)	2.73 (<0.01)	-2.2 (<0.01)	-2.98 (<0.01)
ZNF391	3.07×10^-10^	6:27342394-27371683	Brain, skin, blood, lung	2.54 (0.268)	3.83 (<0.01)	-3.86 (<0.01)	3.13 (<0.01)
ESR2	1.42×10^-9^	14:64550950-64804830	Lung, skin, blood		-5.09 (0.256)	-3.96 (0.243)	-5.58 (0.998)
MTHFD1	5.20×10^-9^	14:64854749-64926722	Lung, blood, skin		-3.05 (<0.01)	-3.18 (0.018)	-3.32 (<0.01)
MEI1	9.03×10^-10^	22:42095503-42195460	Brain, skin, lung, blood	6.52 (0.424)	5.88 (<0.01)	5.95 (<0.01)	6.37 (0.042)
XPNPEP3	2.65×10^-8^	22:41253081-41363838	Blood, skin, lung, brain	3.97 (<0.01)	4.87 (0.045)	5.11 (0.017)	5.42 (0.035)
CCDC134	2.14×10^-9^	22:42196683-42222303	Brain, lung, blood, skin	3.91 (<0.01)	1.63 (<0.01)	2.49 (<0.01)	-1.82 (<0.01)
DESI1	1.10×10^-9^	22:41994032-42017100	Lung, skin, brain, blood	2.35 (<0.01)	1.51 (<0.01)	2.34 (<0.01)	2.91 (<0.01)

### Knowledge-Based Analysis

A total of 23 out of the 27 pleiotropic protein-coding genes have been identified in previous GWASs on depression or ADs (**Supplementary Table 6**). Among these 23 genes were 16 genome-wide risk genes for depression, including *BEND4*, *DENND1A*, *ESR2*, *L3MBTL2*, *NEGR1*, *PCDHA1*, *PCDHA2*, *PCDHA3*, *PCDHA4*, *PCDHA5*, *PCDHA6*, *RBX1*, *SLC30A9*, *SORCS3*, *SYNE2*, and *TMEM161B*, and 8 genome-wide risk genes for ADs, including *BOLL*, *DIAPH1*, *GLB1*, *MOB4*, *NEK6*, *OVOL1*, *RANGAP1*, and *RBX1*. Enrichment of the 27 genes in the GWAS catalog-reported genes revealed that these genes were enriched in several mental disorders and basophil neutrophil counts, as well as neutrophil counts, supporting the involvement of these genes in neurodevelopmental conditions and atopic diseases (**Figure 2C** and **Supplementary Table 7**).

**Figure 4:**
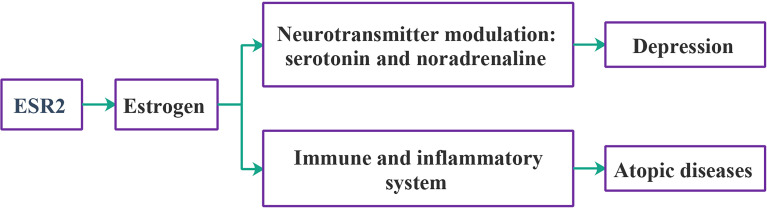
Schematic relationships of *ESR2* with depression and ADs.

PPI analysis showed that a majority of the 82 genes are interconnected, forming one large network and several small networks (**Supplementary Figure 2**). Schematics of *ESR2* gene interactions with depression and ADs are shown in **Figure 4**.

## Discussion

In this study, we detected a significant genetic correlation between MDD and ADs (r = 0.18), at a level comparable to that for a previously reported correlation of MDD and autism spectrum disorder (r = 0.16) (34). Our results indicate a much higher polygenicity of MDD when compared to ADs, with substantial polygenic overlap between these conditions identified. Nearly 90% of causal variants influencing the risk of ADs may also affect MDD. Cumulative evidence supports a close relationship between these two conditions in the context of underlining genetics.

More importantly, causal relationships between MDD and ADs were discovered. In particular, a major causal effect of genetic liability to depression on ADs was detected. Although liability to ADs also exerts a statistically significant causal effect on MDD, the size of this effect is relatively small (b = 0.05). Previous studies already showed the possible influence exerted by MDD on ADs. For instance, patients with MDD show elevated levels of non-esterified fatty acids in plasma (35); other studies showed that fatty acids may contribute to the development of atopic diseases such as hay fever and asthma (36). Elevated serum interferon levels may contribute to eczema and also are commonly detected in MDD (37). Moreover, MDD has been shown to stimulate the production of cytokines (38), including IL-13 and IL-6, both of which are also strongly involved in asthma pathogenesis (39). Our findings are consistent with these previous studies and partially explain the previously reported comorbidity of MDD and ADs (8–12), while adding novel insights into underlying pathogenetic mechanisms. Notably, one previous study reported that depression may lead to asthma rather than the opposite (40). The causal effect of ADs on MDD should be further evaluated in additional datasets.

Shared genetic liability between MDD and ADs offers the possibility of employing polygenic risk scores (PRS) for evaluating allergic risks in MDD patients and the risk of developing depression in AD patients. This strategy may lead to an improvement in the clinical management of these conditions. Shared biological markers of MDD and ADs are far from being well studied. The cross-trait analysis revealed that MDD and ADs share 18 loci and a panel of protein-coding genes. The majority of these pleiotropic protein-coding genes have been previously implicated either in depression or in ADs, with a genome-wide significance level. For example, the *RBX1* gene was reported as a significant contributor to both depression (41) and ADs (42). To shed new light on the genetic susceptibility of ADs and MDD, we have concentrated on the estrogen receptor β encoding gene *ESR2* for further discussion.

Estrogen is capable of modulating neurotransmitter turnover to enhance the levels of serotonin and noradrenaline and participates in the regulation of serotonin receptor amounts and function (43). Accumulating evidence indicates the involvement of estrogen signaling in depression (44). In females, estrogen fluctuations are associated with depressed mood (45), and the beneficial effects of estrogen-containing hormone treatments were reported (46, 47). The gene for estrogen receptor β, *ESR2*, has been previously identified as a genome-wide significant gene contributing to MDD (18, 48). As the levels of estrogen are easily modulated by pharmacological means, the association between *ESR2* and MDD may inform the development of personalized treatment modalities for this condition. Notably, model studies in neonatal rats treated with antidepressant clomipramine uncovered both the changes in the levels of estrogen receptors on the surface of brain cells and the neurochemical changes that resemble human depression (49). The role of estrogens in the development of ADs is noticeable as well. Women have a higher prevalence of asthma and display its greater severity than men (50). Estrogen receptors are found on numerous immune-regulatory cells, with estrogen-dependent responses favoring the shift toward allergy. In particular, estrogens promote allergic response by stimulating Th2 polarization, boosting class switching of B cells to IgE production, and prompting mast cell and basophil degranulation (51). *ESR2* and its product, estrogen receptor β, have been suggested as potential targets for asthma treatment (52). There is also accumulating evidence supporting estrogens’ role in hay fever and eczema (53, 54). In particular, there is a correlation between the mean number of ER-β-positive cells in the nasal mucosa and seasonal allergy symptoms (55).

This study identified *ESR2* as a novel genome-wide significant contributor to ADs, providing strong support for the involvement of the estrogen pathway in ADs. Fine-mapping of TWAS had assigned the posterior probability for causality for *ESR2* in the skin, blood PBMCs, and lung tissue at 0.998, 0.256, and 0.243, respectively. Although the fine-mapping of TWAS hits did not support the involvement of *ESR2* in the brain, analysis of existing literature points at its role in neurodevelopment and mental disorders. Together, our findings highlight *ESR2* as a critical gene for both MDD and ADs and point to its relevance at the therapy target.

The presented study has several strengths. First, we utilized the largest combination of available datasets as a study backbone. Furthermore, to avoid potential population heterogeneity across the studies, we limited our analysis to individuals of European ancestry. Lastly, the genetic relationship between MDD and ADs was explored systemically by employing multiple analytic frameworks.

However, several limitations should also be noted. The datasets employed in this study only contained data of three subtypes of ADs. Further studies using more datasets covering other subtypes of ADs are warranted to evaluate the associations between MDD and ADs. In TWAS, the gene expression levels are imputed from weighted linear combinations of SNPs and, therefore, may report noise. As our analysis was limited to a genetic component of each trait, hence, the presented results should be interpreted cautiously, with the understanding that human traits arise from a complex web of interactions of various psycho-social-environmental factors.

In summary, our findings reveal shared genetic liability and causal links between MDD and atopic diseases, which may underline the phenotypic relationship between MDD and ADs. Presented results may have implications both for the therapy and for the management of MDD and ADs.

## Data Availability Statement

Publicly available datasets were analyzed in this study. These data can be found here: https://www.ebi.ac.uk/gwas.

## Author Contributions

FZ contributed to the study design and data analysis. HC, SL, and AB contributed to drafting and revising the work. All authors contributed to the article and approved the submitted version.

## Funding

This work was supported by the National Natural Science Foundation of China (81471364).

## Conflict of Interest

The authors declare that the research was conducted in the absence of any commercial or financial relationships that could be construed as a potential conflict of interest.

## Publisher’s Note

All claims expressed in this article are solely those of the authors and do not necessarily represent those of their affiliated organizations, or those of the publisher, the editors and the reviewers. Any product that may be evaluated in this article, or claim that may be made by its manufacturer, is not guaranteed or endorsed by the publisher.
