# Supplementary material for: Shared Genetic Liability Between Major Depressive Disorder and Atopic Diseases
Source: Front Immunol. 2021 Sep 8;12:665160. doi: 10.3389/fimmu.2021.665160 (PMC8455950; doi:10.3389/fimmu.2021.665160)
Supplement: Supplementary file 1 [file DataSheet_1.doc]

**Supplementary File**

1. Supplementary Figure 1. Quantile-quantile plots of the observed meta-analysis statistics versus the expected statistics for MDD and atopic diseases.

2. Supplementary Figure 2. Protein-protein interaction network.

3. Methods

4. References

**1. Supplementary Figure 1. Quantile-quantile plots of the observed meta-analysis statistics versus the expected statistics for MDD and atopic diseases**


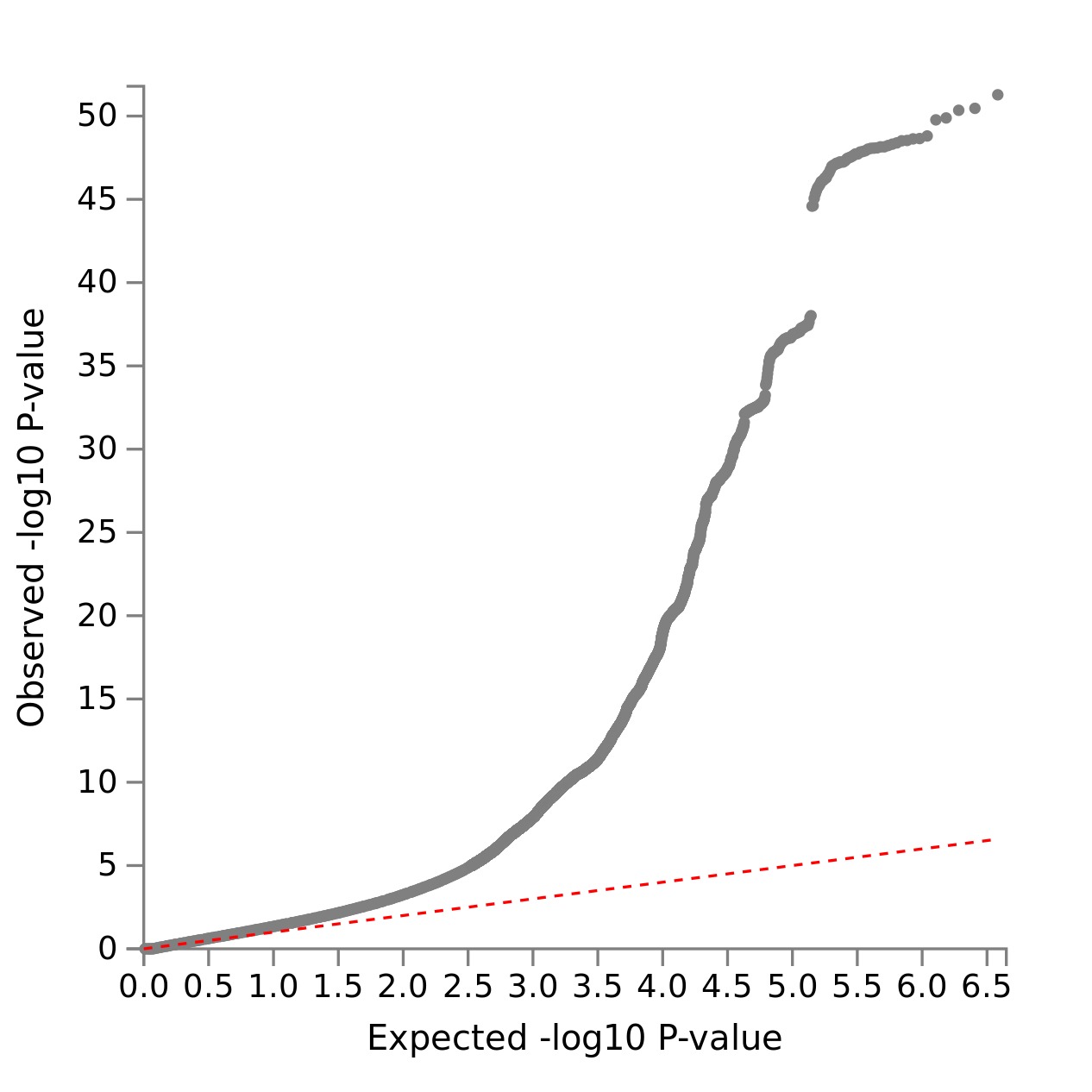


**2. Supplementary Figure 2. Protein-protein interaction (PPI) network.** PPI analysis showed that most of the 82 genes are interconnected.


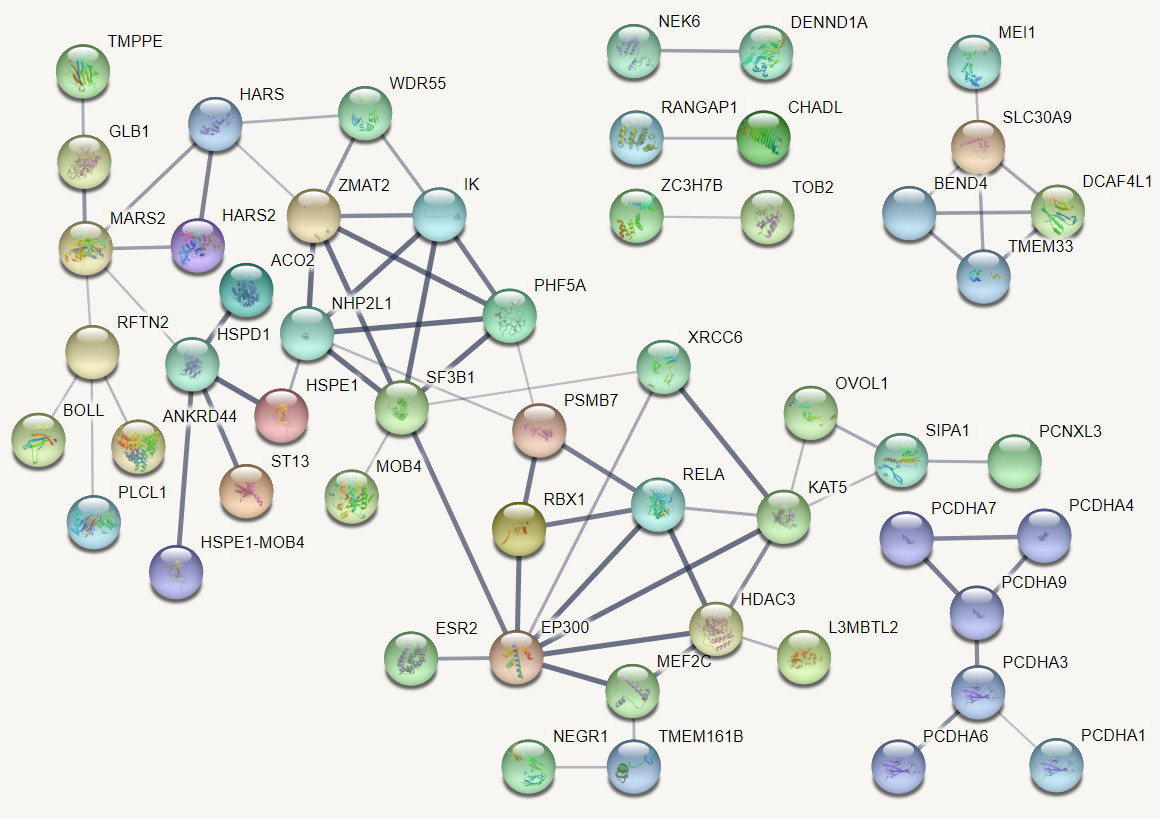


**3. Methods**

**Dataset description**

The MDD dataset includes 135,458 cases and 344,901 controls from seven case-control cohorts. A total of 44 loci were identified as associated with major depression. The first cohort included 29 case-control samples of European descent where lifetime diagnosis of major depressive disorder was ascertained using structured clinical interviews (DSM-V, ICD-9, or ICD-10), clinician-administered checklists, or review of medical records. Six additional cohorts of European ancestry, including the Hyde et al. study (23andMe, Inc.), determined case status using other methods including national or hospital treatment registers, self-reported symptoms or treatment by a medical professional, or direct interviews.

Ferreira et al. performed a genome-wide association study (180,129 cases and 180,709 controls, n = 360,838, all of European ancestry) on a broad atopic diseases phenotype (presence of any one of these three diseases) and identified 136 independent risk variants associated with atopic diseases [1]. The dataset used in our analysis includes results for the meta-analysis of 12 (out of 13) contributing studies. It does not include the 23andMe study; largest sample size per SNP is N=242,569, comprising 96,794 cases and 145,775 healthy controls. Prior to the meta-analysis, association results for each study were adjusted for the respective LD-score regression intercept. The LD-score intercept of the 12-study meta-analysis was 1.022. For each dataset, an additive genetic model was performed in individuals of European descent who reported suffering from asthma and/or hay fever and/or eczema (case group) against those who never reported suffering from any of these three conditions (control group).

Prior to the meta-analysis, standard quality control filters were applied to results from individual studies. After quality control, and restricting the analysis to SNPs present in at least the two largest studies (UK Biobank and 23andMe, Inc.; combined n = 256,623), results were available for 8,307,659 variants, of which most (89%) were available in >95% of the overall sample. Intercept estimates from LD score regression analysis [2], which reflect inflation of test statistics likely due to technical biases, ranged between 1.00 and 1.16. Results from individual studies were adjusted for the observed inflation by multiplying the square of the standard error of each genetic effect estimate by the respective LD score regression intercept. METAL [3] was used to combine association results across studies using an inverse-variance-weighted, fixed-effects meta-analysis. P values from the meta-analysis were further adjusted for the meta-analysis LD score regression intercept of 1.04. The genome-wide significance threshold was set at 3 × 10^-8^, as suggested previously for GWAS analyzing variants with minor allele frequency (MAF) ≥ 1% [4].

**LD score regression**

LD score regression software v1.0.1 were used to analyze the genetic correlation of MDD with atopic diseases. The 1000 Genome project phase 3 [5] were used to estimate the LD structure for European populations, which was obtained from the LD score regression website [2,6,7]. SNPs were filtered by 1.1 million variants, subset of 1000 Genomes and HapMap3 [8], with MAF above 0.05, MHC and other long-range LD regions excluded.

**Polygenic overlap analysis**

Frei et al. introduced a novel statistical framework (MiXeR) to quantify polygenic overlap irrespective of genetic correlation between traits [9]. In MiXeR, a causal mixture model [9] [10]is used to estimate the percentage of variance explained by genome-wide significant SNPs as a function of sample size. For each SNP, i, MiXeR models its additive genetic effect of allele substitution, β_i_, as a point-normal mixture,
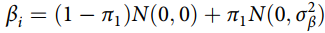
, where π_1_ represents the proportion of non-null SNPs (polygenicity) and σ^2^_β_ represents variance of effect sizes of non-null SNPs. Then, for each SNP, j, MiXeR incorporates LD information and allele frequencies for 9,997,231 SNPs extracted from 1000 Genomes Phase 3 data [2,6,7] to estimate the expected probability distribution of the signed test statistic,
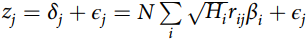
, where N is sample size, H_i_ indicates heterozygosity of ith SNP, r_ij_ indicates allelic correlation between *i*th and *j*th SNPs, and ϵ_j_ ~ N(0, σ^2^_0_) is the residual variance. Further, the three parameters, π_1_, σ^2^_β_, and σ^2^_0_, are fitted by direct maximization of the likelihood function. In the MiXeR analysis, an effective sample size of Neff = 4/(1/Ncase + 1/Ncontrol) was used to account for imbalanced numbers of cases and controls. The python_convert (v0.9.2) pipeline was used to harmonize GWAS summary statistics (https://github.com/precimed/python_convert).

**Cross-trait meta-analysis**

ASSET meta-analysis is an agnostic approach that generalizes standard fixed-effects meta-analysis by allowing a subset of the input GWASs to have no effect on a given SNP. The method exhaustively explore all possible subsets of ‘‘non-null’’ GWAS inputs within a fixed-effect framework to identify the strongest association signal in both positive and negative directions. Only SNPs that were present for both the traits were retained as inputs to the ASSET meta-analysis, resulting in 7,653,691 SNPs for subsequent analysis. We carried out one-sided analysis by exploring models in which all non-null studies have effects in the same direction. Default parameters were applied with the ‘‘h.traits’’ function in ASSET.

FUMA was used to map SNPs to genes and identify LD-independent genomic regions [11]. Nearest genes and functional consequence of each SNP on gene functions were annotated based on ANNOVAR [12]. Genomic risk loci were identified by merging lead SNPs if they were closer than 250 kb apart. Clumping procedures were carried out on the basis of the European 1000 Genomes Project phase 3 reference panel. Due to extensive LD, the MHC region was merged into one region (chr6:25-35Mb). Genes within 100 kb of each variant were mapped.

**MR analysis**

To infer credible causal associations between MDD and atopic diseases, we performed Mendelian randomization analysis using GSMR V1.0.9 [13]. This method utilizes summary-level data to test for putative causal associations between a risk factor (exposure) and an outcome by using independent genomewide significant SNPs as instrumental variables as an index of the exposure. Some methods have been proposed to assess the sensitivity of an MR analysis to detect pleiotropy [14]. These methods, however, do not account for possible LD between SNPs nor the sampling errors in the estimated effect sizes of the instruments on the exposures. The GSMR uses heterogeneity in dependent instrument (HEIDI) to test for heterogeneity in b_xy_ estimated at multiple correlated instruments [15]. GSMR extends this approach to detect heterogeneity in b_xy_ estimated at m near-independent instruments (the method accounts for remaining LD not removed by clumping). The basic idea is to test where there is a significant difference between b_xy_ estimated at an instrument i (i.e., b_xy_(i)) and b_xy_ estimated at a target SNP that shows a strong association with the exposure. The power of detecting heterogeneity increases with the strength of association between the target SNP and exposure. HEIDI outlier detection was used to filter genetic instruments that showed clear pleiotropic effects on the exposure phenotype and the outcome phenotype. We used a threshold P value of 0.01 for the outlier detection analysis in HEIDI, which removes 1% of SNPs by chance if there is no pleiotropic effect. We tested for bidirectional causation by repeating the analyses while switching the role of each correlated phenotype as an exposure and intelligence as the outcome. For each trait, we selected independent (r^2^ = 0.1), genome-wide significant lead SNPs as instrumental variables in the analyses.

The method estimates a putative causal effect of the exposure on the outcome (b_xy_) as a function of the relationship between the SNP’s effects on the exposure (b_zx_) and the SNP’s effects on the outcome (b_zy_), given the assumption that the effect of non-pleiotropic SNPs on an exposure (x) should be related to their effect on the outcome (y) in an independent sample only via mediation through the phenotypic causal pathway (b_xy_). The estimated causal effect coefficients (b_xy_) are approximately equal to the natural log odds ratio for a case-control trait. An odds ratio of 2 can be interpreted as a doubled risk compared with the population prevalence of a binary trait for every standard deviation increase in the exposure trait.

**Fine-mapping of TWAS associations**

FOCUS (Fine-mapping Of CaUsal gene Sets) is software to fine-map transcriptome-wide association study (TWAS) statistics at genomic risk regions. The software takes as input summary GWAS data along with eQTL weights and outputs a credible set of genes to explain observed genomic risk. FOCUS v0.6.10 was used to identify potential causal genes from the meta-analysis result of MDD and AD [16]. A multiple tissue, multiple eQTL reference panel weight database from the software (https://github.com/bogdanlab/focus/) was used. This combines GTEx v7 weights from PrediXcan [17] with METSIM, NTR, YFS, and CMC weights from FUSION software [18] into a single usable database for FOCUS. FUSION requires GWAS summary statistics (Z-scores) and eQTL weights as input. By integrating the GWAS summary result, expression weights, and LD among SNPs, it identifies causal gene to be included in a 90%-credible set and give a posterior probability (PIP) to estimate the causality in relevant tissues. We ran FOCUS on the TWAS data using prior variance σ_c_^2^ = 80 and the prior probability for a gene to be causal is set to p = 1 × 10^-3^.

We applied FOCUS analysis on the meta-analysis result of MDD and AD in four relevant tissues, including brain, blood, lung, and skin. The results from the four tissues were merged and irrelevant tissues other than the four tissues were removed. The results were filtered by adjusted TWAS P value (FDR < 0.05). Results from various regions of a tissue were merged into one by retaining the one with highest TWAS-Z value.

**4. References**

1 Ferreira MA, Vonk JM, Baurecht H, Marenholz I, Tian C, Hoffman JD, et al. Shared genetic origin of asthma, hay fever and eczema elucidates atopic diseases biology. Nat Genet. 2017;49(12):1752-57.

2 Bulik-Sullivan BK, Loh PR, Finucane HK, Ripke S, Yang J, Schizophrenia Working Group of the Psychiatric Genomics C, et al. LD Score regression distinguishes confounding from polygenicity in genome-wide association studies. Nat Genet. 2015;47(3):291-5.

3 Willer CJ, Li Y, Abecasis GR. METAL: fast and efficient meta-analysis of genomewide association scans. Bioinformatics. 2010;26(17):2190-1.

4 Fadista J, Manning AK, Florez JC, Groop L. The (in)famous GWAS P-value threshold revisited and updated for low-frequency variants. Eur J Hum Genet. 2016;24(8):1202-5.

5 Genomes Project C, Auton A, Brooks LD, Durbin RM, Garrison EP, Kang HM, et al. A global reference for human genetic variation. Nature. 2015;526(7571):68-74.

6 Bulik-Sullivan B, Finucane HK, Anttila V, Gusev A, Day FR, Loh PR, et al. An atlas of genetic correlations across human diseases and traits. Nat Genet. 2015;47(11):1236-41.

7 Finucane HK, Bulik-Sullivan B, Gusev A, Trynka G, Reshef Y, Loh PR, et al. Partitioning heritability by functional annotation using genome-wide association summary statistics. Nat Genet. 2015;47(11):1228-35.

8 Altshuler DM, Gibbs RA, Peltonen L, Dermitzakis E, Schaffner SF, Yu F, et al. Integrating common and rare genetic variation in diverse human populations. Nature. 2010;467(7311):52-58.

9 Frei O, Holland D, Smeland OB, Shadrin AA, Fan CC, Maeland S, et al. Bivariate causal mixture model quantifies polygenic overlap between complex traits beyond genetic correlation. Nat Commun. 2019;10(1):2417.

10 Holland D, Frei O, Desikan R, Fan CC, Shadrin AA, Smeland OB, et al. Beyond SNP heritability: Polygenicity and discoverability of phenotypes estimated with a univariate Gaussian mixture model. PLoS Genet. 2020;16(5):e1008612.

11 Watanabe K, Taskesen E, van Bochoven A, Posthuma D. Functional mapping and annotation of genetic associations with FUMA. Nat Commun. 2017;8(1):1826.

12 Wang K, Li M, Hakonarson H. ANNOVAR: functional annotation of genetic variants from high-throughput sequencing data. Nucleic Acids Res. 2010;38(16):e164.

13 Zhu Z, Zheng Z, Zhang F, Wu Y, Trzaskowski M, Maier R, et al. Causal associations between risk factors and common diseases inferred from GWAS summary data. Nat Commun. 2018;9(1):224.

14 Burgess S, Bowden J, Fall T, Ingelsson E, Thompson SG. Sensitivity Analyses for Robust Causal Inference from Mendelian Randomization Analyses with Multiple Genetic Variants. Epidemiology. 2017;28(1):30-42.

15 Zhu Z, Zhang F, Hu H, Bakshi A, Robinson MR, Powell JE, et al. Integration of summary data from GWAS and eQTL studies predicts complex trait gene targets. Nat Genet. 2016;48(5):481-7.

16 Mancuso N, Freund MK, Johnson R, Shi H, Kichaev G, Gusev A, et al. Probabilistic fine-mapping of transcriptome-wide association studies. Nat Genet. 2019;51(4):675-82.

17 Gamazon ER, Wheeler HE, Shah KP, Mozaffari SV, Aquino-Michaels K, Carroll RJ, et al. A gene-based association method for mapping traits using reference transcriptome data. Nat Genet. 2015;47(9):1091-8.

18 Gusev A, Ko A, Shi H, Bhatia G, Chung W, Penninx BW, et al. Integrative approaches for large-scale transcriptome-wide association studies. Nat Genet. 2016;48(3):245-52.
